# Full-Quantum Treatment of Molecular Systems Confirms Novel Supracence Photonic Properties

**DOI:** 10.3390/ijms24087490

**Published:** 2023-04-19

**Authors:** Wei Wan, Alexander D. Q. Li

**Affiliations:** Department of Chemistry, Washington State University, Pullman, WA 99164, USA

**Keywords:** supracence, full-quantum, fluorescence, photonic, up-conversion, two-photon

## Abstract

Our understanding of molecules has stagnated at a single quantum system, with atoms as Newtonian particles and electrons as quantum particles. Here, however, we reveal that both atoms and electrons in a molecule are quantum particles, and their quantum–quantum interactions create a previously unknown, newfangled molecular property—supracence. Molecular supracence is a phenomenon in which the molecule transfers its potential energy from quantum atoms to photo-excited electrons so that the emitted photon has more energy than that of the absorbed one. Importantly, experiments reveal such quantum energy exchanges are independent of temperature. When quantum fluctuation results in absorbing low-energy photons, yet still emitting high-energy photons, supracence occurs. This report, therefore, reveals novel principles governing molecular supracence via experiments that were rationalized by full quantum (FQ) theory. This advancement in understanding predicts the super-spectral resolution of supracence, and molecular imaging confirms such innovative forecasts using closely emitting rhodamine 123 and rhodamine B in living cell imaging of mitochondria and endosomes.

## 1. Introduction

**Figure 1:**
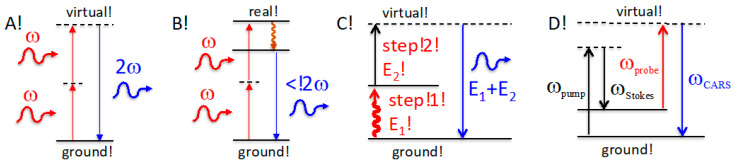
Second harmonic generation (**A**), two-photon fluorescence (**B**), anti-Stokes Raman (**C**), and coherent anti-Stokes Raman (**D**) involve 2 or more steps in excitations; thus, all are nonlinear processes; their response depends on a single input quadratically or more than one inputs.

One fascinating phenomenon observed in 1961 was when red light of a ruby laser was frequency doubled into an ultraviolet beam [1]. Later the birth of nonlinear optics established basic principles for such infrared-in, yet green-light-out phenomena as second harmonic generation (SHG). The “mechanism” of second harmonic generation involves the virtual energy transition of two simultaneous photons that are converted exactly into a single doubled-frequency photon (Figure 1A) [2]. SHG responses are typically ultrafast (femtosecond or less), but they do require the material to have no inversion symmetry [3]. Molecules or materials emitting more energy than that of input is very intriguing because of the curiosity about how a more energetic photon is born from multiple low energetic photons. Until now, only a few processes are known to convert energy from multiple low-energy inputs into one output with more energy than that of a single input. Here, we compare and contrast these phenomena to our work, from which the significance and broader impact emerge.

Unlike SHG, two-photon fluorescence (TPF) results in a real transition and excited state; energy is partially lost, and as a result, the energy of an emitted photon is less than the sum of two excitation photons, but higher than a single excitation photon (Figure 1B). Two-photon fluorescence has practical benefits, including deeper tissue penetration of the long-wavelength excitation light and highly confined focal spots, where two photons strongly and spatially overlap [4,5,6]. For example, DAPI dye (4′,6-diamidino-2-phenylindole) is universally used to stain DNA, but requires cell-damaging ultraviolet excitation. Similarly, small endogenous NADH (nicotinamide adenine dinucleotide hydride) and serotonin are very difficult to use for one-photon excitation in the deeper ultraviolet (e.g., 250 nm). However, a two-photon fluorophore can be excited in visible or near-infrared [7]. Again, TPF is a nonlinear optical process that requires two-photon simultaneous excitation and TPF intensity depends on the excitation intensity (*I*_EX_) quadratically [8].

When molecules start their optical transition from an excited vibrational state rather than the lowest-energy ground state, the emission wavelength could be shorter than the absorption wavelength, but the *total* emitted energy is still not greater than the total absorbed energy. There are two hypotheses in this category: anti-Stokes Raman [9,10] and anti-Stokes fluorescence [11,12,13]. Both are not new and should be described as nonlinear optical processes because at room temperature molecules essentially occupy the lowest vibrational state and the hypothesis requires two excitation steps, some reported two-step excitations are heat and photon, to promote them to the excited state.

In anti-Stokes Raman, the first excitation may be caused by a previous photon followed by relaxation to a vibrationally excited ground state. The second excitation results in anti-Stokes scattering via a virtual state (Figure 1C). Anti-Stokes scattering is much weaker than Stokes Raman because it is a two-step, nonlinear process [14]. Accordingly, coherent anti-Stokes Raman (CARS) intensity depends on the power of excitation photons, including pump, Stokes, and probe (Figure 1D), because it uses two photons to populate an excited ground state, followed by a third probe-photon excitation. Thus, CARS involves three excitation steps, and thus a third-order process [15,16].

**Figure 2:**
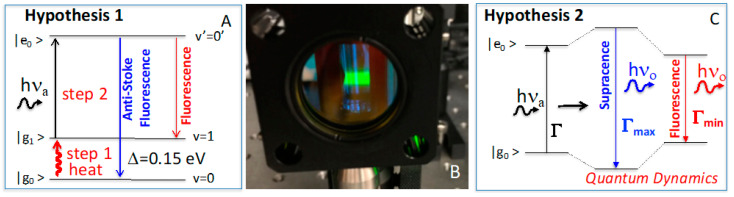
(**A**) Hypothesis 1 uses two-step excitation to produce higher-frequency emission: heat and photon. (**B**) Experimental observation of higher-frequency green photons created by lower-frequency yellow-green photons. (**C**) Hypothesis 2 uses full-quantum systems, in which electron–atom entanglement induces quantum dynamics and causes the expansion and contraction of the electronic transition energy, thus emitting higher-frequency photons at suitable conditions.

The definition of anti-Stokes clearly states a second-order, nonlinear process involving two excitation steps. Thus far, the principle of anti-Stokes fluorescence almost exclusively uses heat as the first excitation step, the so-called hot band [17,18], and photon as the second excitation step (Figure 2A). This anti-Stokes fluorescence principle defines itself as a two-step, nonlinear process. Thus, anti-Stokes fluorescence intensity must increase when both (1) heat or temperature increases and (2) photo-excitation power increases. Specifically, anti-Stokes fluorescence intensity should be pseudo-first order to photonic power and should increase as the temperature rises according to Boltzmann distribution. Together, anti-Stokes fluorescence is, in fact, a second-order process because its intensity depends on the product of both heat energy or temperature and photonic power.

Temperature affects many physical properties such as quantum yields and extinction coefficients. Currently, testing of the anti-Stokes hypothesis as a two-step, nonlinear phenomenon does not include the variation in these temperature-dependent parameters. Thus, such variable-temperature anti-Stokes fluorescence measurements are meaningless without proper calibrations to these parameters that are also sensitive to temperature.

In summary, none of the thus far reported phenomena consider that the molecules donate their internal energy to the emitted photons. All the above processes including anti-Stokes fluorescence are nonlinear, and thus require multiple excitation steps (Figure 1); the total output energy is equal to or less than, but not above, the total input energy.

However, we recently discovered an above-excitation emission, [19,20] hereafter named supracence; when a yellow-green laser excites rhodamine B, higher-frequency green light radiates strongly (Figure 2B). Supracence is independent of temperature; thus, it is a linear process or requires only a single step of photoexcitation based on previous data [19,20] and work reported here (Figure 2C). This is exciting because, for the first time, the molecules donate their internal energy to the emitted photons, thus emitting higher-energy photons. Moreover, for the first time, the total output energy is greater than the total input energy, with the molecules contributing the difference and recuperating energy through other processes. Therefore, supracence is an exciting molecular phenomenon, from which exciting applications will emerge.

## 2. Results and Discussion

### 2.1. Temperature Independent Supracence Excitation and Emission

Most anti-Stokes fluorescence reports provide neither validation of the pseudo-first-order to photo-excitation power (*I*_EX_) nor heat energy or temperature (*T*) dependence. Others validate the pseudo-first-order to light power but did not confirm how heat or temperature is involved. Some reports plotted the anti-Stokes intensity versus temperature, but they did not consider that extinction coefficient and quantum yield, which are instrumental to emission brightness, also depend on temperature. Because of these, the literature on anti-Stokes fluorescence has been in a state of confusion currently, not entirely certain whether to treat it as second order or somewhere in between first and second order.

(1) $$I_{AS}=k_{\begin{array}{l} 1 \end{array}}I_{EX}\frac{e^{-\varepsilon_{1}/kT}}{\sum_{v=0}^{N}e^{-\varepsilon_{v}/kT}}$$

(2) $$\frac{I_{AS}}{\varepsilon (T)\Phi (T)}=k_{2}I_{EX}\frac{e^{-\varepsilon_{1}/kT}}{\sum_{v=0}^{N}e^{-\varepsilon_{v}/kT}}$$

In light of our own work on supracence, we came across two reports [12,21] that claimed (1) anti-Stokes fluorescence was used for laser cooling and (2) rhodamine 101 in *acidified ethanol* satisfied a nonlinear process and its anti-Stokes fluorescence intensity increased as a function of both excitation light (*I*_EX_) and temperature (*T*). Among all anti-Stokes fluorescence reports, these were the best description of the excitation process involving two steps and two variables, thus second order. Their putative theory of anti-Stokes intensity as a function of both excitation power and temperature is summarized in Equation (1), thus a nonlinear process, because two variables (*I*_EX_ and *T*) were assumed to contribute to the excitation, where *v* = 0, 1, 2, …N, and *k* is Boltzmann constant, *T* is temperature, and ε_i_ is the i-th energy level. Immediately after their publication, Mungan and Gosnell commented and concluded that anti-Stokes laser cooling was impossible under their experimental conditions [22]. Meanwhile, our supracence work raises the objection of whether the observed effect was indeed anti-Stokes fluorescence. Not only was the temperature-dependence of quantum yield and extinction coefficient not considered in anti-Stokes laser cooling, but the *acidification of ethanol* will also alter the equilibrium significantly between rhodamine spirolactone and its ring-open form. It is well-known that acid will catalyze reversible ring-opening and ring-closure in rhodamine dyes [23]. Therefore, the temperature-dependence of equilibrium shift will significantly change the concentration of the ring-open rhodamine emitter, hence yielding a temperature-dependent emission intensity. Without proper calibration of temperature-dependence of quantum yields and extinction coefficients and, more importantly, consideration of acid-catalyzed ring-opening and ring-closure that affect absorption and emission, the asserted observation of anti-Stokes fluorescence is unmeaningful and misleading.

**Figure 3:**
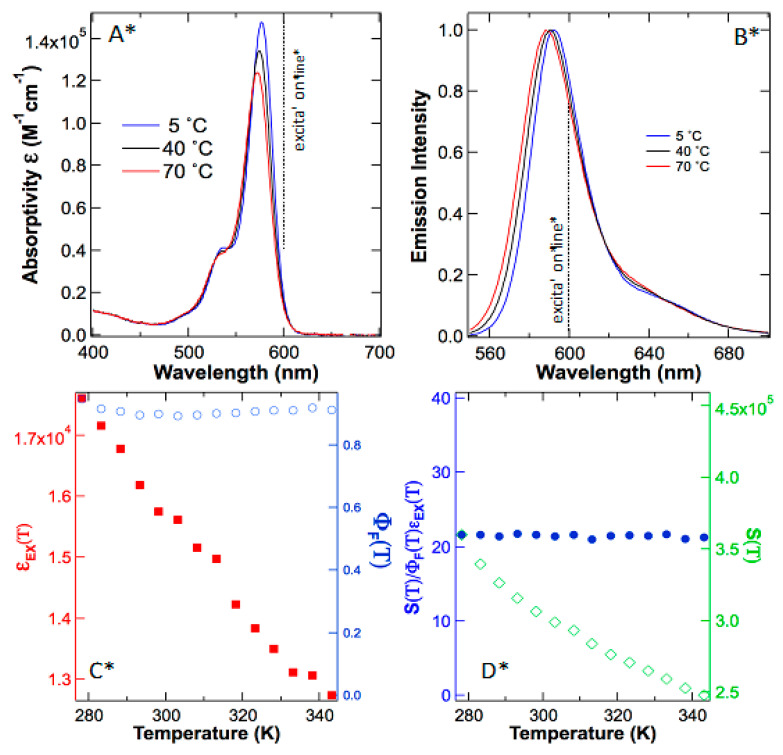
Absorption (**A**) and emission (**B**) spectra of sulforhodamine 101 in ethanol are both functions of temperature. Because the absorption peak maximum leans toward blue as the temperature rises, the extinction coefficient at excitation line (600 nm) decreases as temperature rises (red squares in (**C**)). Meanwhile, quantum yields change slightly at various temperatures (blue circles in (**C**)). As a result, the supracence maximum at ~590 nm plummets as temperature rises (green diamonds in (**D**)). However, after calibrations by quantum yields and extinction coefficients, the normalized supracence intensity is independent of temperature (blue circle in (**D**)).

To find the truth, we repeat the previously reported “anti-Stokes fluorescence” using sulforhodamine 101 in ethanol without acidification. As depicted in Figure 3A,B, both absorption and emission spectra of sulforhodamine 101 have subtle changes as the temperature rises. These results reveal that extinction coefficient and quantum yield are both a function of temperature and any variable temperature experiments must be calibrated using these parameters before reaching further conclusions about temperature effects on emission intensity.

Next, we plotted the extinction coefficient ε(T) change against temperature and use these data to calculate the quantum yields Φ(T) at various temperatures of sulforhodamine 101 (Figure 3C), which are in good agreement with what was reported earlier [24]. Because both quantum yield and extinction coefficient are temperature dependent, true anti-Stokes fluorescence must be calibrated against these influences by improving the superficial model described by Equation (1) to the adequate Equation (2). Only after removing the influences by temperature-dependent quantum yields and temperature-dependent extinction coefficients, can the temperature-promoted, vibrationally excited population in |g_1_〉 state be investigated. Here, the ground state wavefunction is |g_0_〉 ≈ |φ_g_〉|0〉 and the vibrationally excited but electronic ground state is |g_1_〉 ≈ |φ_g_〉|1〉. The lowest excited state wavefunction is |e_0_〉 ≈ |φ_e_〉|0〉; |φ_g_〉 and |φ_e_〉 are electronic ground and excited wavefunctions, respectively, and |0〉 is the lowest vibrational wavefunction of the atoms while |1〉 is the first vibrationally excited state. Accordingly, the appearance and growth in |g_1_〉 population should contribute *I*_AS_ in a manner like a concentration increase, which is distinct from influences by extinction coefficient and quantum yield that physically alter transition probabilities.

Finally, to determine the supracence peak maximum intensity at 588–592 nm, we excite sulforhodamine 101 in ethanol at 600 nm. Contrary to what was reported in rhodamine 101, our results on sulforhodamine 101 illustrated an opposite trend; supracence *S*(*T*) peak intensity plummets as the temperature rises (Figure 3D). The major reason for the peak maximum to abate is that molar extinction coefficient ε(T) is diminishing due to the hypsochromic peak leaning in the absorption spectrum (Figure 3A). After calibration to the temperature effects of extinction coefficient and quantum yield, the emission peak intensity reveals a zero-order dependence on temperature (Figure 3D). In other words, anti-Stokes fluorescence did not occur in sulforhodamine 101. What was observed is supracence that obeys Equation (3), independent of temperature (n = 0).

(3) $$\frac{I_{Supra}}{\varepsilon (T)\Phi (T)}=k_{3}{I^{m}}_{EX}T^{n}=k_{3}I_{EX}\;(m=1,\;n=0)$$

**Figure 4:**
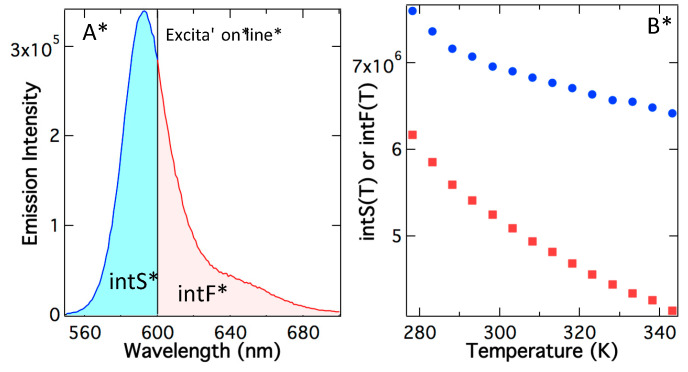

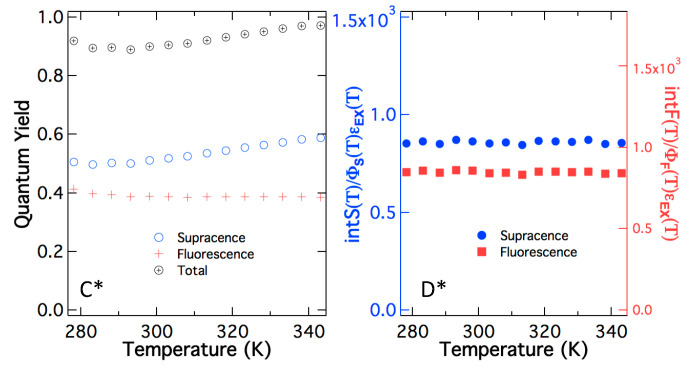
(**A**) The emission spectrum is divided by excitation line into supracence area (intS) and fluorescence area (intF). Such integrated supracence intensity (blue circles) and integrated fluorescence intensity (red squares) are plotted in (**B**). Both are plummeting such as the peak maximum S(T) in Figure 4. (**C**) Quantum yields using the total area under the emission curve Φ_T_ are divided into supracence quantum yield Φ_S_ and fluorescence quantum yield Φ_F_. (**D**) After calibrating to variations in quantum yields and extinction coefficients, the normalized integrated intensities for both supracence and fluorescence remain constant as temperature varies. Thus, temperature plays no role in both supracence and fluorescence excitation (n = 0 in Equation (3)).

The above analysis uses peak maximum, but a more detailed analysis should use the integrated photons under the emission curve. In Figure 4A, the excitation line divides the total emission into integrated supracence area (intS) and integrated fluorescence area (intF). As temperature rises, both the fluorescence area and supracence area decrease, although at different rates (Figure 4B). The main reason for the slow reduction in the supracence area is hypsochromic shifting in the emission spectrum (Figure 3B). As a result, at a higher temperature, supracence gains more share of the total emitted photons. This prediction is validated in Figure 4C, in which quantum yields as a function of temperature are depicted. The top curve is the total quantum yields at various temperatures determined using the total area under the emission curve. The area-determined quantum yields agree with the peak-maximum quantum yields (Figure 3C); both show little change via temperature. The total quantum yield (Φ_T_) is the sum of fluorescence quantum yield (Φ_F_) and supracence quantum yield (Φ_S_), Φ_T_ = Φ_F_ + Φ_S_. It is apparent that supracence quantum yields are increasing as the temperature rises, but fluorescence quantum yields are diminishing as the temperature rises. The reason is that at the fixed excitation line, the total emission spectrum is blue shifting, slicing a larger and larger share for supracence at the expense of fluorescence.

If anti-Stokes fluorescence were the underpinning mechanism, the supracence area after calibrating quantum yield and extinction coefficient would rise as the temperature rose, obeying Equation (2). If supracence is the underpinning mechanism, the supracence area after calibrating quantum yield and extinction coefficient will be constant or independent of temperature, obeying Equation (3). The experimental results in Figure 4D reveal that the calibrated supracence area has zero-order to temperature (n = 0). In conclusion, after removing temperature effects by quantum yields and extinction coefficients, both supracence peak maximum and supracence emission area are independent of temperature, demonstrating that heat plays no role in supracence excitation.

Figure 4D also shows that the fluorescence area after calibration of quantum yields and extinction coefficients also remains constant as temperature varies. Supracence is a new phenomenon and less is known about its excitation order, but fluorescence has been well known and its first-order photoexcitation does not depend on temperature. The fact that fluorescence and supracence behave the same way toward temperature variation states that both excitations have no heat involvement. Thus, both supracence and fluorescence are independent of, and zero-order to, temperature.

The crucial merit in determining linear supracence or nonlinear anti-Stokes fluorescence is that first-order phenomena are much more sensitive and intense than second-order phenomena, typically orders of magnitude greater (compare Equation (2) to Equation (3)). Thus, supracence is expected to have broader impacts in technological applications than that of second-order anti-Stokes fluorescence because the absolute intensity and sensitivity of supracence will be orders of magnitude higher than those of a nonlinear, two-step process.

**Figure 5:**
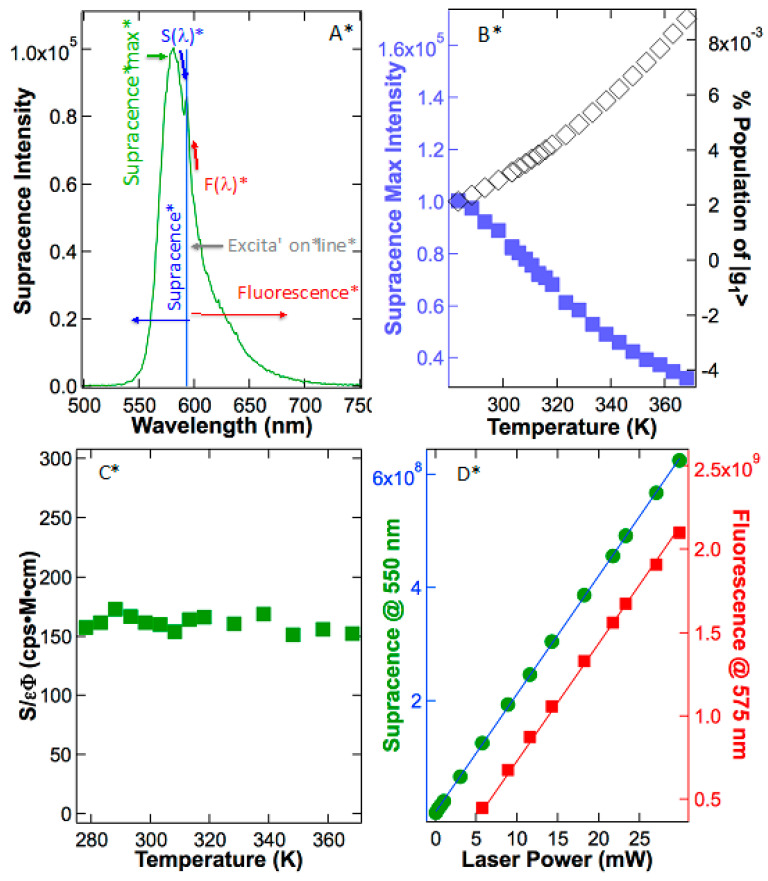
(**A**) Rhodamine B was excited at 595 nm so that its supracence peak maximum at 581 nm can be unambiguously determined. (**B**) The supracence peak maximum intensity at 581 nm is plummeting against absolute temperature (blue squares). Boltzmann distribution of putative |g_1_〉 population (black diamonds) plotted against temperature predicts growth from 0.21% to 0.88% (283 to 368 K), but the peak maximum did not match such a growth, as evidenced in (**C**). Supracence intensities normalized by variable-temperature quantum yields and extinction coefficients reveal zero-order dependence on temperature. Thus, heat is not involved in supracence excitation. (**D**) Supracence intensity (green circles) and fluorescence intensity (red squares) scale linearly with the 561 nm laser excitation powers. Thus, both are one-step excitation, first-order, and linear processes.

The above analysis of anti-Stokes fluorescence provides the framework to test hypotheses 1 and 2 again and again. In Figure 5A, rhodamine B was excited at 595 nm, and strong emission with a peak at 581 nm, a higher frequency than that of excitation, is observed. Again, do the emitted photons with higher frequency than that of excitation photons belong to anti-Stokes fluorescence or supracence? If the above-excitation emission were anti-Stokes fluorescence due to absorbing heat energy, the observed intensity would increase as the supplied heat energy increased. As demonstrated in Figure 5B, supracence intensity did not increase when the solution temperature was increased. Rather, the supracence intensity plummeted as the temperature rose primarily because temperature-induced quenching reduced the dye’s quantum yield. In sulforhodamine 101, the extinction coefficients decrease as the temperature rises mainly causes the reduction in the supracence peak. In rhodamine B, however, quantum yields play the major role in the abatement of supracence peak intensity at elevated temperatures (Figure 5B).

Following the anti-Stokes mechanism, molecules must *slowly* absorb heat obeying Boltzmann distribution. This will not keep up with ultrafast photon excitation, but still offers the molecule a putative way to hot band |g_1_〉 once in a long while. For typical rhodamines, the experimental measured vibrational level difference between |g_0_〉 and |g_1_〉 in an electronic ground state is Δ = 0.15 eV. According to Boltzmann distribution, anti-Stokes fluorescence should curve upward, mirroring the population or concentration increase in |g_1_〉 in Figure 5B (black diamonds) as described in Equation (2), where Δ = ε_1_ − ε_0_ = 0.15 eV. Had the molecules recovered slowly via Boltzmann equilibrium, the calibrated anti-Stokes fluorescence intensity would have steadily increased from 283 to 368 K by ~4.2 times because the concentration of the “hot molecules” were more than quadrupled. In reality, the emission response does not change at all after calibrating to quantum yield and extinction coefficient changes (Figure 5C; green squares). Therefore, Boltzmann distribution analyses also confirm that higher energy emission does not originate from the hot band |g_1_〉 or anti-Stokes fluorescence. However, temperature independence does support supracence, as illustrated in hypothesis 2 (n = 0).

Specifically, hypothesis 2 does not consider heat as an excitation step; it only considers a single photon excitation as described by Equation (3). Figure 3D, Figure 4D and Figure 5C proved zero-order dependence on temperature, justifying no involvement of temperature during excitation. Figure 5D reveals that there is a linear relationship between the supracence intensity at 550 nm versus the excitation power at 561 nm (m = 1). This linear relationship is crucially important because it proves that only a single photon is involved in the excitation. In fact, the behavior of the supracence resembles very much like the manner of fluorescence in Figure 5D, which is also a single-photon-excitation phenomenon. These data prove that supracence is zero-order to temperature (n = 0) and first-order to photoexcitation (m = 1), fitting the supracence mechanism described by Equation (3), and disputing the anti-Stokes mechanism.

### 2.2. Phantom Theoretical Absorption Peak-Shape

Thus far, all reports on anti-Stokes fluorescence follow the same belief: molecules somehow absorb heat and become vibrationally excited, which are then undergoing below-gap-energy absorption. Specifically, all anti-Stokes fluorescence requires a 1→0 vibronic excitation or absorption, i.e., an electro-optical transition from |g_1_〉 to |e_o_〉, in which |g_1_〉 is a “hot” state because of vibrationally absorbing heat. For dyes such as rhodamines, it is slightly challenging to identify where these vibronic bands are, but it is abundantly obvious for fused-ring aromatic compounds such as perylene diimide [25,26,27].

**Figure 6:**
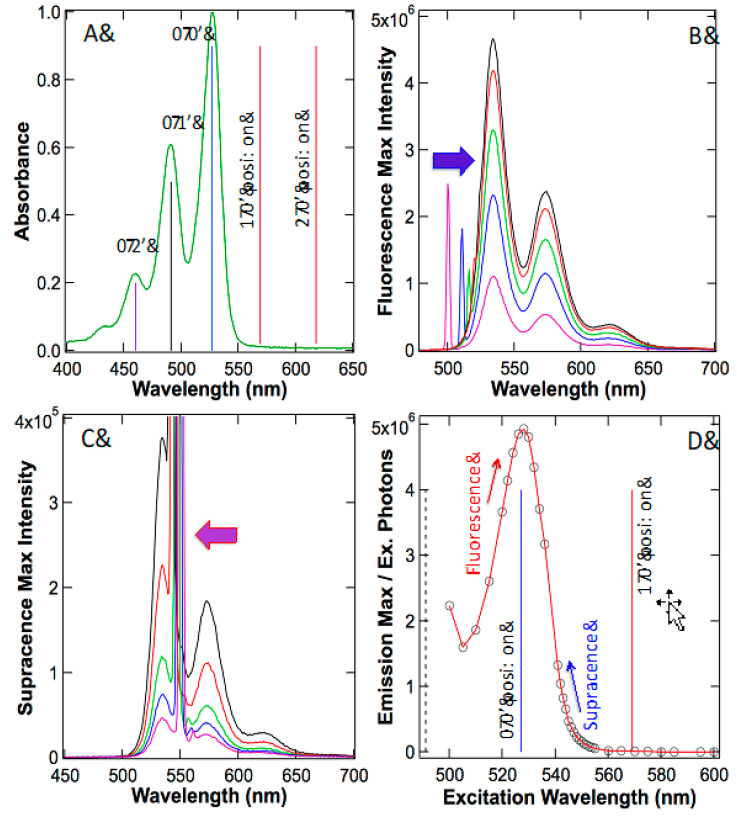
(**A**) Perylene diimide has clearly resolved vibronic bands so that the hot band 1→0 position is unambiguously identified at 569 nm, but there is no such absorption peak at this position. (**B**) As excitation laser lines approach the 0→0 absorption (pink→blue→green→red→black), the fluorescence intensity grows. (**C**) As excitation laser lines approach the 0→0 absorption in the opposite direction (pink→blue→green→red→black), the supracence intensity rises. The big arrows in (**B**) and (**C**) point out the laser excitation lines. (**D**) Plot of emission maximum per unit of excitation photons reveals that both fluorescence and supracence excitations pump energy into the same absorption band—the 0→0 transition. Note that at the 1→0 position, there is no resonance whatsoever. In fact, in between 0→0 and 1→0, supracence profile is much greater than that at the 1→0 position, where anti-Stokes resonance should occur. This did not happen, thus disputing anti-Stokes.

Figure 6A illustrates the absorption spectrum of perylene diimide, and in particular, the emphasis is that the vibronic positions are clearly identified, with 0→0 at 527 nm, 0→1 at 491 nm and 0→2 at 460 nm. According to the anti-Stokes fluorescence mechanism, it must have an absorption peak at the 1→0 position, which is at 569 nm because such absorption is pivotal to downstream anti-Stokes fluorescence. However, experimental data reveal that there is no peak at the 1→0 position of 569 nm, nor at the 2→0 position of 618 nm. The residue absorbance at the 1→0 position is due to the spillover of 0→0 absorption, a tailing effect. These results conclude that all anti-Stokes fluorescence approaches are building on an imaginary absorption—a mirage that was never identified experimentally. The absorption band, which is responsible for all the anti-Stokes fluorescence, simply does not exist.

The above results demonstrated that there is no absorption resonance at the 1→0 position in absorption spectroscopy. Is there an emission resonance at the 1→0 position using emission spectroscopy? Figure 6B illustrates the tunable laser exciting fluorescence maximum at 534 nm; as the laser excitation wavelength approaches the 0→0 position (527 nm), the fluorescence maximum intensifies. Similarly, yet in the opposite direction, Figure 6C illustrates the supracence peak maximum at 534 nm. When the laser excitation line approaches the 0→0 position of 527 nm, the supracence maximum strengthens. Along the path to the 0→0 position, the supracence laser excitation line passes the 1→0 position at 569 nm. According to the anti-Stokes fluorescence theory, there must be a resonance at the 1→0 position because it is the resonance position for anti-Stokes fluorescence and the whole anti-Stokes intensity depends on it. In reality, as shown in Figure 6D, there is no emission resonance when excitation arrives at the 1→0 position of 569 nm, either.

In fact, when both fluorescence and supracence are converging to the 0→0 position from opposite directions, the emission responds with maximizing intensity (Figure 6D). Corollary to these observations is that both supracence and fluorescence are exciting into the same 0→0 transition as proved earlier using the full quantum model [19,20], in which electrons and atoms are treated as two sub-quantum systems or dual quantum systems. Nevertheless, how does low-energy light excite into the higher 0→0 electro-optical transition to create high-energy supracence?

### 2.3. A Single Classical Structure versus Myriad Quantum Structures

**Figure 7:**
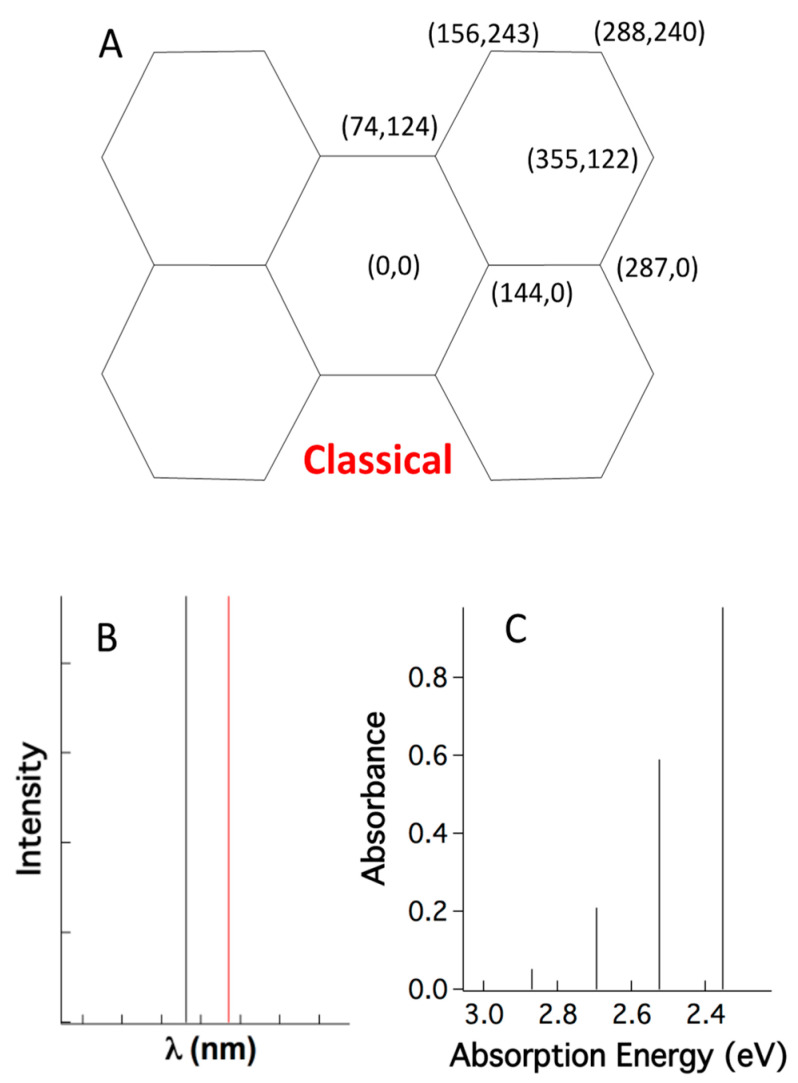
(**A**) Fixing the coordinates of the atoms in electronic quantum calculations of perylene is a Newtonian treatment for atoms and (**B**) results in stick-absorption (black) and emission (red) spectra. (**C**) When an electronic absorption stick is coupled with vibrational energy levels, a single stick line in (**B**) split into multiple stick lines following Franck–Condon Factors.

The enigma is that, as yet, no known linear optical processes create output photons with more energy than that of input photons. The reason that current understanding cannot rationalize supracence is that it drops the dual quantum realism and treats atoms in molecules as non-quantum particles. The quantum treatment of vibrating atoms stands alone and does not interact and integrate with the electronic quantum results. This way, quantum chemistry typically fixes the coordinates of atoms (Figure 7A) and determines the quantum behavior of the electrons. Thus, the atoms with fixed positions are treated as classical Newtonian particles, while the electrons are treated as quantum particles. In other words, the vibrational wavefunction of atoms |υ〉 is reduced to a pair of classical coordinates (x, y), thus ignoring quantum structural dynamics. This classical-quantum hybrid treatment yields bizarre stick spectra for electronic absorption (Figure 7B, black stick) and emission (Figure 7B, red stick). After calculating vibrational levels and electronic levels separately, and then simply adding them together, the “coupled” vibronic absorption spectrum follows the Franck–Condon progression [28], but still comprises sticks, unlike experimentally broad and smooth peaks (Figure 7C).

Thus far, the hybrid treatment (Newtonian atoms mixed with quantum electrons) of molecules or a single-quantum system has typically been applied in explaining experimentally observed peaks in electronic spectra using many congested rotational levels associated with electronic transition [29]. However, dyes in a solid state do not rotate and their absorptions are still not sticks [30,31]. This puzzling disparity leads to another explanation of sample inhomogeneity [32,33], but the unique peak shape must originate from a matching inhomogeneous sub-species population, yet there is no such evidence. Importantly, one single molecule has no inhomogeneity and, thus, single-molecule emission has no inhomogeneous issues; however, single-molecule spectra still uniquely shape into broad peaks, not sticks [34,35,36,37].

**Figure 8:**
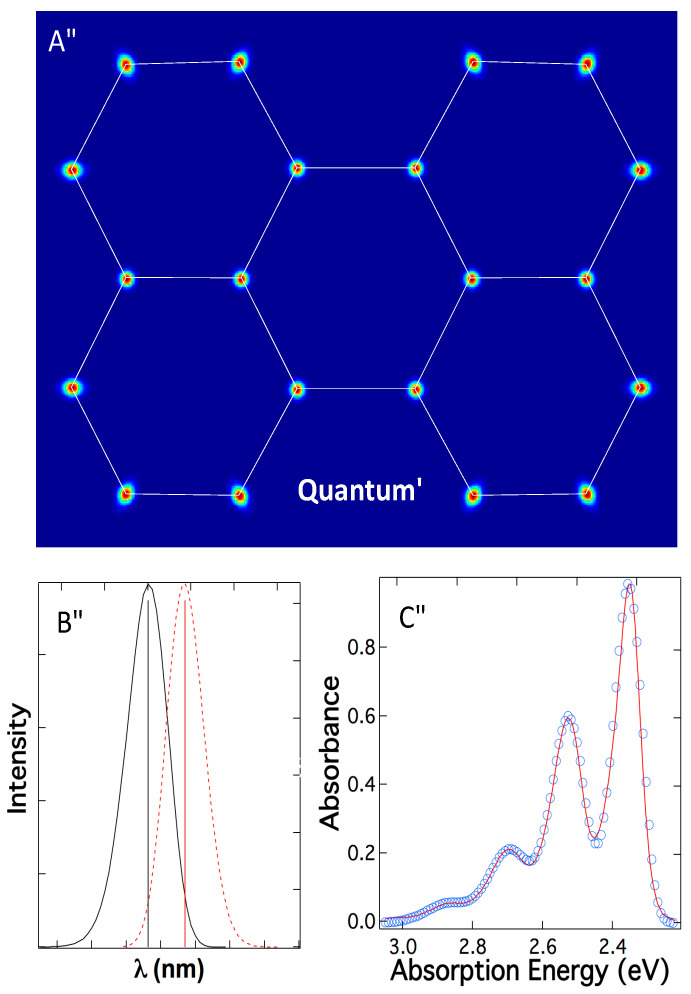
(**A**) Trapped in potential wells, atoms are quantum particles with probability orbitals rather than fixed points as described classically in Figure 7A; (**B**) these probability orbitals create a smooth peak shape that depicts how the probability of a molecular structure is related to its absorption (black) and emission (red) energy—creating realistic spectral peaks, not sticks. (**C**) Coupling the smooth peak shape created by quantum atoms in (**B**) with Franck–Condon Factors of perylene diimide in Figure 7C successfully predicts the shape of its absorption spectrum (circles: theory; line: experiment).

Experimental data prove hypothesis 2 is correct, but what is the underpinning foundation for this full (electron and atoms) quantum dynamic model? In fact, an atom trapped by chemical bonds is a quantum particle in an ellipsoid potential well and must be treated quantum mechanically so that vibrational quantum levels emerge. Mixing Newtonian mechanics and the Schrodinger equation is physically meaningless. In full quantum treatment, the atoms in their molecule no longer have fixed classical positions, rather they are moving in ellipsoidal quantum orbitals (Figure 8A and Movie S1). For example, atoms with symmetric bonds (center ring of perylene) have quantum orbitals that are nearly spherical, resembling much of the electronic s-orbital. Atoms without symmetric bonds, such as periphery carbons of perylene, have orbitals that are ellipsoidal due to asymmetry (Figure 8A). These are quantum orbitals describing the positional probability of the atoms and should not be confused with conventional “atomic orbitals” that describe electrons.

Applying full quantum mechanics to both electrons and atoms results in two sub-quantum systems for electrons φ_g_ and atoms υ, respectively, but these two sub-quantum or dual quantum systems are not independent; rather they (φ_g_ and υ) are entangled [38]. In any molecule, lighter quantum electrons, φ_g_, are entangled, coupled, and integrated with the heavier quantum atoms, υ, resulting in convoluted phenomena and, hence, intricate optical spectra. In such full quantum systems, the energy levels of the heavy quantum particles or atoms remain moderately discrete and fluctuate due to potential limitations, but lighter quantum particles or electrons constantly change their energy levels within a narrow distribution to satisfy the quantum dynamics of the atoms and molecular potential energy restricting the atoms. Such dynamic electronic energy levels predict that absorption and emission must disperse from a single line into uniquely structured peaks (not sticks) according to the quantum effects of the atoms (Figure 8B). This means both ground states and excited states have energy level distribution rather than a single discrete level, thus rationalizing both fluorescence and supracence will occur.

The quantum interaction slates that the probability of vibrational transitions of the heavier quantum atoms is convoluted with the broad, intricate electronic absorption of the lighter quantum electrons (Figure 8B), thus yielding realistic vibronic absorption and emission spectra (Figure 8C: circles) in excellent agreement with experimental observation (Figure 8C: line). Applying Fermi’s Golden rule to both electron wavefunction, φ_g,_ and atom wavefunctions υ results in the observed vibrational transition between different vibrational states, which is governed by Franck–Condon factors (Figure 8C) with characteristics of the progressive envelope, i.e., |〈0|0〉|^2^ > |〈1|0〉|^2^ > |〈2|0〉|^2^, etc. [28,39]. The validation of intricate peak shape enlighteningly endorses that full quantum treatment fundamentally underpins molecular absorption, emission, and supracence.

**Figure 9:**
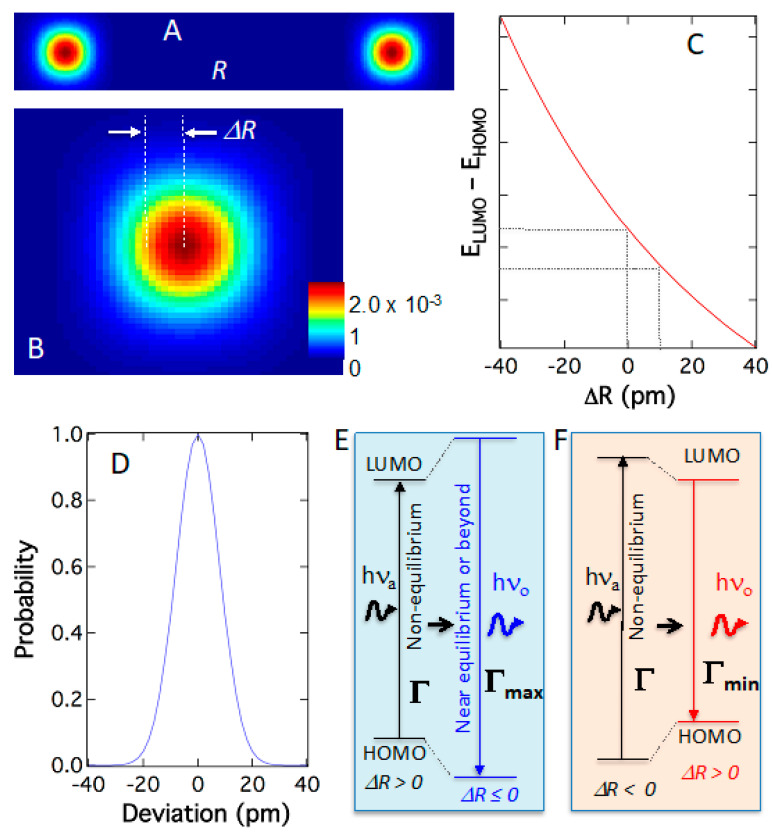
(**A**) The simplest two-atom full-quantum approximation: electron–atom quantum interaction predicts the probability of each atom with an enlarged view (**B**) to show bond length elongation due to quantum fluctuation of the atoms (**C**) As the bond undergoes quantum dynamics, the electronic transition energy of absorption or emission (*E*_LUMO_–*E*_HOMO_) changes as a function of the molecular structure or ΔR. At bond-stretched structures or Δ*R* > 0, the absorption gap is smaller, but the molecule is strained. Near-equilibrium structures (Δ*R* ≈ 0), the relaxed molecule has a larger emission gap. (**D**) Plotting the atom’s probable position against Δ*R* yields a realistic peak shape that can be used to describe experimental spectra. (**E**) Absorbing low-energy photon in a strained quantum structure (Δ*R* > 0) and emitting at relaxed quantum structures (Δ*R* ≈ 0) will produce supracence—a quantum photon like a tensed bow shooting a physical arrow. Conversely, absorbing high-energy photon in a relaxed structure (Δ*R* ≈ 0) and emitting at tensed structures (Δ*R* > 0) yields fluorescence (**F**).

Of particular importance is the quantum interaction energy caused by the dynamics of the dual sub-quantum systems of atoms and electrons. Here, we will write the general Hamiltonian as a sum of three parts (Equation (4)), where ${\hat{H}}_{A}$ is the Hamiltonian for atoms, ${\hat{H}}_{e}$ is the Hamiltonian for electrons, and ${\hat{H}}_{Ae}$ is the interaction Hamiltonian between atoms and electrons [40,41]. To compute the quantum interaction energy, we use the Born–Oppenheimer or adiabatic ansatz |φ_g_(t)〉|υ(t)〉 and plug into the Born rule. The conclusion explicitly reveals that the quantum interaction energy, $\langle {\hat{H}}_{Ae}\rangle \left(t\right),$ is dynamically evolving as a function of time, even if a non-entangled state, |φ_g_(t)〉|υ(t)〉, is used [38]. This energy exchange between atoms and electrons is depicted in Figure 2C and Figure 9.

The full-quantum treatment of molecules requires that Schrodinger equations for **both atoms and electrons** within a molecule be solved **simultaneously**, either entangled or non-entangled [19]. Precisely, the lighter quantum particles and their electronic transitions are correlated, concatenated, and entangled to the positions of the heavier quantum particles. The single-quantum system of molecules only solves the electron Schrodinger equation using Newtonian coordinates to describe the atoms, while ignoring entanglement and quantum interaction, thus leaving some physical properties, e.g., absorption intricate peak shape, emission tails, and supracence, dangling in no-man’s land. Considering a full-quantum system for molecules, however, divulges both realistic molecular absorption and emission properties and predicts the existence of novel supracence spectroscopy and microscopy. Because the positions of quantum atoms vary continuously and smoothly in a Gaussian-like fashion, the optical absorption and emission are also shaped into smooth Gaussian-like peaks accordingly.

(4) $$\hat{H}={\hat{H}}_{A}+{\hat{H}}_{e}+{\hat{H}}_{Ae}$$

(5) $$\langle \hat{H}\rangle \left(t\right)=\langle \upsilon \left(t\right)\left|{\hat{H}}_{A}\right|\upsilon \left(t\right)\rangle \langle \varphi_{g}\left(t\right)\left|\left|\varphi_{g}\left(t\right)\rangle +\langle \upsilon \left(t\right)\right|\right|\upsilon \left(t\right)\rangle \langle \varphi_{g}\left(t\right)\left|{\hat{H}}_{e}\right|\varphi_{g}\left(t\right)\rangle +\langle \varphi_{g}\left(t\right)\left|\langle \upsilon \left(t\right)\left|{\hat{H}}_{Ae}\right|\varphi_{g}\left(t\right)\rangle \right|\upsilon \left(t\right)\rangle =\;=\langle {\hat{H}}_{A}\rangle \left(t\right)+\langle {\hat{H}}_{e}\rangle \left(t\right)+\langle {\hat{H}}_{Ae}\rangle \left(t\right)$$

The full quantum treatment of molecular systems using adiabatic ansatz can be explicitly solved [42]. A simple harmonic potential well could be used for the molecular ground state and Morse potential could be used for an excited state potential well, both work well because they produce emission and absorption peaks matching experimental spectra. One important conclusion replaces the Newtonian coordinates with an atom probability map, such as in Figure 8A. Another important conclusion is that the quantum energy of the atoms and the quantum energy of the electrons are constantly exchanging while time elapses, as evidenced in Equation (5). Molecular potential energy exchange atom quantum energy, which in turn exchange with electronic energy. These energy flows dominate within the molecule. Because optical absorption and emission are ultrafast; at typical emission rates, heat transfer is insufficient to keep up with the above energy flows. As such, ultrafast absorption and emission and full quantum theory simply rule out anti-Stokes fluorescence or heat involvement during a repeated optical transition.

In the full quantum model, using Born–Oppenheimer approximation, and further isolating each atom of an *N*-atom molecule in its potential well, there are *N* sub-Schrodinger equations for atoms, but there are only 3*N*–6 independent vibrational energies/frequencies for a non-linear molecule, because when all *N* atoms are moving in synchrony, the whole molecule is translating or rotating along an axis, the quantum effect disappears and the molecule simply follows Newtonian mechanics, drifting, diffusing, or spinning. These irreducible 3*N*–6 vibration frequencies correspond to the conventional normal modes but are here regarded as molecular phonons because it is more logical considering their energy exchange within the molecular system. Each phonon has a characteristic vibration frequency ($\langle {\hat{H}}_{A}\rangle \left(t\right)=A_{vib}$), which may be either infrared or Raman active, but it also stores or releases internal potential energy (*P*) by varying the barriers of limiting potential wells, which manifests as the amplitude of atomic displacements (x, y, or z): *P_k_* = ½(*k*_x_x^2^ + *k*_y_y^2^ + *k*_z_z^2^), i.e., the changes in bond length during quantum dynamics (Figure 2C and *vide infra*).

Precisely, the full quantum treatment of molecules manifests as the lighter quantum system of electrons riding on the heavier quantum system of atoms. The parallel example in Newtonian mechanics is a motorcycle traveling on top of a moving train. One would reach surreal conclusions about the motorcycle had the motion of the train been ignored. Because experimentally atoms for the most part retain their identity in molecules, full quantum theory describes molecules more realistically than dual quantum sub-systems using Born–Oppenheimer approximation, which is still more realistic than single-quantum models that do not update the origins of electron orbitals according to the position of the quantum atom, such as describing the motorcycle speed without considering the train. As such, the electronic energy levels are determined by not only the electrons but also by the atoms, whose quantum movement cannot be ignored. For example, Newtonian–quantum hybrid treatment of a dye fixes the positions of all conjugate p-orbitals, thus placing supracence in no-man’s land. However, the full quantum principle recognizes that each p-orbital is riding on a quantum atom and constantly “dancing” according to the probability produced by the orbital of the atom, υ (see Movie S2). Note, atoms are treated quantum mechanically, not nuclei. The electron orbitals (for example, p orbitals above) are convoluted by the orbital of atoms unlike single-quantum models, in which the electron orbital is not convoluted. In such concatenated quantum systems, absorption and emission automatically shape into intricate peaks and supracence emerges naturally.

Proof of the quantum behavior of atoms comes from the infrared and Raman vibrational spectroscopy experimentally. Had atoms in a molecule behaved according to classical Newtonian mechanics, infrared and Raman spectra would not exist, because classical particles do not have quantized energy levels, hence absorbing and emitting light. Another proof of quantum atoms originates from experimental vibronic bands in absorption (Figure 6 and Figure 8C). Electronic absorption having fine vibronic structures also concludes that atoms in molecules behave according to dual sub-quantum systems, rather than Newtonian particles plus a single electronic quantum system.

### 2.4. Full Quantum (FQ) Molecules Make All Pieces Fall into Place

Because the lighter quantum system of electrons and the heavier quantum system of atoms, namely, molecular phonons, are entangled, coupled, concatenated, and bonded, together they produce vibronic bands due to strong electron–phonon coupling. This coupling goes beyond simply adding the energy levels that are solved separately, but rather each move of the atoms or phonons creates a different transition energy for the electrons. While this is under-appreciated, other correlations due to the dual interacting quantum systems have not broadly been studied thus far. FQ theory predicts that many properties may be correlated in a predictable way. The two properties concerning supracence, for example, are bond elongation (structural variation) produced by the quantum effect of atoms and transition energy in absorption or emission spectra determined by the electronic states. Here, we show that the bond elongation and transition gap are correlated or entangled, such that a change in one will result in a predictable trend in the other.

Figure 9A depicts the simplest model of two-atom in simplified full quantum treatment, where each atom distributes according to its wavefunction squared (probability), not classical coordinates. The reason for the simplest model is that current computation power is not adequate for calculating a single quantum system and consequently full quantum computations for dyes are beyond current computation capacity. Nonetheless, the full quantum model leads us in the correct directions. When the quantum atom elongates the bond or Δ*R* > 0 according to its quantum probability (Figure 9B), the electronic energy levels change; accordingly, particularly the absorption-or-emission gap or transition energy *E_LUMO_–E_HOMO_* diminishes (Figure 9C). Thus, as the quantum atoms dance in their orbitals following pre-determined quantum probability [υ]^2^, the transition energy disperses into a peak-shaped distribution around their most probable positions as shown in Figure 9D. These phenomena originate from quantum–quantum effects intrinsically and are independent of temperature or other perturbations. Such a correlated relationship reveals that the atomic position and electronic transition are strongly correlated. Therefore, bond elongation correlates to the shrinkage of electronic transition energy for absorption or emission and *vice versa*. This demonstrates that the quantum dynamics of the atoms will result in spectral dispersion in absorption and emission. Importantly, this means that there are multiple energies for absorption or emission, all within a narrow distribution of energy. Some absorb more energy and emit less, while others absorb less and emit more energy. For example, absorbing at elongated bonds and emitting at shortened bonds will result in supracence (Figure 9E), and conversely, fluorescence (Figure 9F).

Relevant to our discussions are three forms of energies within a molecule: total electronic bonding energy ($E=\langle {\hat{H}}_{e}\rangle \left(t\right)+\langle {\hat{H}}_{Ae}\rangle \left(t\right)$), total phonon vibrational energy ($A=\langle {\hat{H}}_{A}\rangle \left(t\right)$) from 3*N*-6 molecular phonons, and the total internal potential energy (*P*) stored within all chemical bonds and their molecular structure that forms the potential wells for holding the atoms in place. Energy conservation or the thermodynamic first law requires their sum or total energy to be constant in adiabatic conditions without photo-absorption or emission (*E* + *A* + *P* = constant). In full quantum systems, the quantum movements of the heavier system are dictating the system, intrinsic and inalienable, maintaining a constant energy (*A*); thus, the first FQ law is that **the total electron-bonding energy (*E*) of the lighter quantum system must fluctuate** due to such motion of heavier quantum particles. Experimentally, such fluctuation is the origin of the absorption or emission intricate peak shape instead of a single-quantum stick. The fluctuating electronic total energy seminally advances our understanding of current static energy minimum that yields a fixed molecular structure. Thus, the single-quantum system yields a single classical molecular structure, but the electron–atom dual-quantum systems predict not a single molecular structure, rather a series of quantum dynamic structures with a smooth distribution.

In view of FQ molecules, energies (*E*, *A*, or *P*) can freely exchange. Potential energy (*P*) from a molecular structure and bonds set up the potential wells for the atom to behave quantum mechanically and determine its vibrational energy (*A*). Moreover, the quantum movement of the atoms is constantly changing the energy landscape for the electrons (*E*) and regulates the energies of electronic orbitals. Naturally and significantly, potential energy (*P*) and quantum energies (*A* and *E*) flow back and forth like current.

Because the total energy of the molecule is constant, the second enlightening FQ principle reveals that the electronic quantum energy must exchange with the molecular internal potential energy in the bonds to maintain the total energy constant, i.e., high *E* with low *P* and *vice versa*. Because phonon energy *A* can be considered as a constant in each state for the molecule before or after photoexcitations, the sum of electron energy *E* and potential energy *P* must be constant (*E* + *P* = constant), too. During photoexcitation, phonon energy *A* is facilitating energy exchange from *E* and *P* or vice versa. The fluctuation of electron energy *E* requires the potential energy *P* to mirror its fluctuation and change accordingly. Otherwise, molecules cannot exist. In a vacuum, for example, a molecule must exchange its total electronic bonding energy with internal energy, because no surrounding functioning as an energy reservoir for it to deposit energy to or to recover energy from. Thus, **the molecule must have a mechanism to store energy internally, in the form of internal potential energy, *P***. Therefore, the correlation between bond elongation and electronic transition gap shrinkage or bond shortening and transition gap expansion is facilitated by the potential energy stored in chemical bonds and molecular structures.

In short, molecular structures or bonds function as an energy reservoir during photoexcitation. Such energy exchange, which originates from the electron and atom dual quantum entanglement, is unique for molecules, and it disappears completely in atomic absorption or emission that is purely a single quantum system. Thus, molecules enable supracence, but single atoms or ions without bonds cannot produce supracence.

When photoexcitation occurs in bond-stretched structures (Δ*R* > 0; far from equilibrium and lower transition energy) and subsequent relaxation brings the molecule to near-equilibrium structures for emission (Δ*R* ≈ 0 and higher transition energy), the molecule will emit a photon with more energy than that absorbed (Figure 9E). This is supracence [19,20]. Considering the photon and the molecule as a thermodynamic system before absorption (*E_A_*, *A_A_*, *P_A_*) or after emission (*E_E_*, *A_E_*, *P_E_*) leads to energy conservation, i.e., *E_A_* + *A_A_* + *P_A_* + *hν_A_* = *E_E_* + *A_E_* + *P_E_* + *hν_E_*, where ν_A_ and ν_E_ are absorbed and emitted photon frequencies, respectively. When the molecule returns to the same quantum state (equilibrium structures for both atoms and electrons) after emission, all quantum energies are canceled due to the same quantum state (*E_A_* = *E_E_* & *A_A_* = *A_E_*). A conclusion expressed in Equation (6) thus emerges, where Δ*ν* = *ν_E_* − *ν_A_*. Supracence hence occurs when Δ*P* = *P_A_* − *P_E_* > 0 and fluorescence occurs when Δ*P* < 0 (Figure 9F). A stretched quantum bond is analogous to a stretched mechanical bow, both building up potential energy in it. The stretched bow releases its potential energy to accelerate an arrow and likewise, the stretched quantum bond releases its strain, through quantum-potential energy exchange (*vide supra*), to emit a high-energy photon or a quantum “arrow” with a different color in the case of supracence. Thus, a quantum–quantum interaction between electrons and atoms unfolds as molecular quantum archery that imparts supracence.

(6) Δν=ΔP/h

One clarification worth mentioning is where does the energy of supracence come from. This question becomes clear when considering typical excitations in which a molecule absorbs multiple photons (M). Some photons result in fluorescence yielding negative Δ*P*, while other photons impart supracence producing positive Δ*P*. Yet, still, non-radiative processes end up negative Δ*P*. Therefore, the sum of all Δ*P* is approximately zero, retaining the molecule in its original ground state (Equation (7)). The molecule functions as an energy reservoir. In summary, supracence gains energy probably at the expense of fluorescence and other energy-related processes.

(7) $$\overset{M}{\underset{i=1}{\sum }}\Delta \nu_{i}=\overset{M}{\underset{i=1}{\sum }}\frac{\Delta P_{i}}{h}=0$$

### 2.5. Super-Spectral Resolution of Supracence Spectroscopy and Imaging—Experimental Validation

The value of a theory resides in its ability to guide experiments. Many theories fail to guide experiments and remain as an after-facts tool to rationalize what was observed. The electron–atom quantum entanglement predicts that the supracence peak will be near-Gaussian-like and narrow, which was validated experimentally in supracence imaging below.

In the electron–atom quantum interaction, the electronic transitions, both absorption and emission, are restricted within the maximum (Γ_max_) and minimum (Γ_min_) gaps between HOMO (E_HOMO_) and LUMO (E_LUMO_) (Figure 2C and Figure 9E,F). The maximum (Γ_max_) and minimum (Γ_min_) transition energies are the results of energy exchange with quantum atoms restricted by the molecular internal potential energies. As demonstrated in Figure 8A, the quantum movements of the atoms are severely limited by molecular potentials, and the energy-gain range available for supracence is rather limited, δΓ = Γ_max_ − Γ_min_. This narrow fluctuation of electronic transition energy (δΓ) originated from the quantum effect of the atoms is compensated by the molecular potential energy (*P*), which fundamentally results in a super-spectral resolution in supracence spectroscopy and imaging.

Only when excitation photons (Γ) have energies in-between maximum (Γ_max_) and minimum (Γ_min_) transition energies, i.e., Γ_min_ < Γ < Γ_max_ (Figure 2C and Figure 9E,F), supracence can occur, because there is no absorption when Γ < Γ_min_, and there is no supracence when Γ > Γ_max_ as the energy gap cannot expand anymore. Anti-Stokes fluorescence would have excited far lower than Γ_min_ using the phantom hot band, while fluorescence will continue to excite the molecule above excitation energy above Γ_max_. Both fluorescence and anti-Stokes fluorescence have no excitation specificity, thus producing broad excitation and emission bands. Supracence, however, has a narrow excitation band and a narrow emission band between Γ_max_ and Γ_min_.

**Figure 10:**
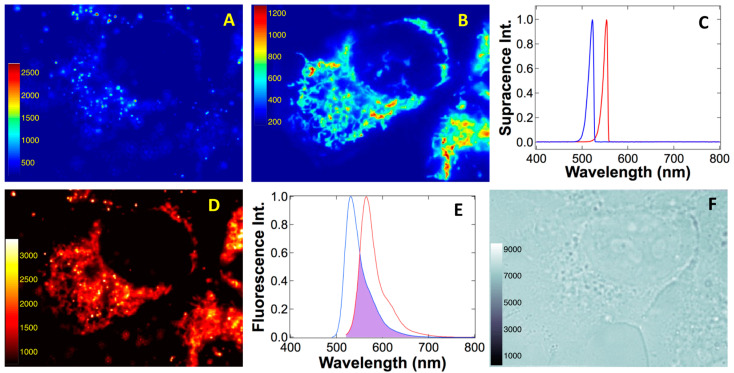
Electron–atom quantum entanglement predicts super spectral resolution. Rhodamine 123 and rhodamine B have absorption and emission maxima only 32 nm apart and are used to label endosomes and mitochondria in live cells. (**A**) Super spectral resolution supracence reveals RhB nanoparticles in endosomes traveling on microtubules, impacting characteristic spot patterns, but Rh123-labeled mitochondria are not observed due to supracence selectivity to RhB. (**B**) Conversely, supracence selectively reveals worm-like, Rh123-labeled mitochondria, but not RhB-labeled spotty endosomes because of super spectral resolution (**C**). However, fluorescence (**D**) cannot resolve these two dyes because it lacks spectral resolution; thus, both spotty endosomes and worm-like mitochondria are mixed together (**E**). Rh123: blue spectra; RhB: red spectra. (**F**) Brightfield image of the cell under study. All image sizes are ~53 × 40 μm^2^.

To demonstrate super-spectral resolution created by electron–atom quantum interaction and entanglement, Figure 10 contrasts supracence spectra and imaging to those of fluorescence. Rhodamine 123 (Rh123) and rhodamine B (RhB) have peak-maximum separation of only 32 nm, but their supracence windows δΓ_Rh123_ and δΓ_Rh123_ practically do not overlap. As a result, we selectively excited RhB within nanoparticles (561 nm) used to label endosomes and lysosomes in living cells [43], but Rh123 supracence in mitochondria was not excited (Figure 10A). Conversely, when we excited Rh123 supracence in mitochondria using 532 nm, no RhB supracence was observed (Figure 10B). Supracence spectroscopy reveals the super-spectral resolution in Figure 10C. Because of the mitochondria worm-like patterns [44,45,46], endosome/lysosome sharp spots [47,48,49,50] can be easily recognized, and they were chosen to corroborate the super spectral resolution. 

Supracence imaging of mitochondria has no measurable crosstalk from endosomes/lysosomes. Moreover, endosomes/lysosomes imaging has no mitochondrial encroachment. Fluorescence imaging at 532 nm, however, reveals both RhB sharp spots of endosomes/lysosomes and Rh123 worm-like patterns of mitochondria (Figure 10D). Not only is fluorescence excitation not selective, but, also, broad fluorescence emissions have a strong overlap in a large spectral region (Figure 10E). These results demonstrate the superiority of supracence at wavelength multiplexing and dyes with absorption or emission peaks separated by only 32 nm that can be effectively resolved during the imaging of a living cell (Figure 10F). 

## 3. Materials and Methods

**Tunable laser measurements of fluorescence and supracence**. Both supracence and fluorescence spectrometers were built using a tunable laser (SuperK, NKT Photonics, Portland, OR) as the excitation source. Unless the excitation wavelengths are fixed wavelengths such as 532 nm or 561 nm, the measurements were carried out using NKT SuperK FIANIUM white laser. The specific wavelength was obtained using an LLTF tunable high contrast filter, which allows a single laser line (<2 nm) to pass with high efficiency. Both supracence and fluorescence were detected at 90-degree angle after filtering the laser excitation scattering and proper attenuation of the emission intensity. The attenuation using multiple neutral density filters was necessary, because they maintain the PMT detector in the dynamic linear response range. Before the detector, a monochromator was installed to resolve the emission spectra.

**Supracence imaging**. The supracence microscope was built using an inverted Olympus X81 system equipped with a high numerical aperture oil immersion objective (PLANO100XO3, 1.45 NA). For supracence imaging, two supracence excitation lasers were used: 561 nm and 532 nm (both from Crystal Laser, Reno, NV, USA). The choice of these two lasers was based on their availability not because they have maximum supracence efficiency. Specifically, the 532 nm laser is not at the maximum supracence efficiency for rhodamine 123, but the 561 nm laser is near the optimum supracence efficiency for rhodamine B. Despite this non-optimized condition, this in-house-built microscope still delivered superior results for supracence imaging. Both lasers were collimated and introduced to the supracence microscope from the back port. The laser rays were reflected to the 100X objective of microscope by two multi-edge dichroic mirrors, ZT405-488/568rpc-UF1 (Chroma, Bellows Falls, VT, USA), and Di03-R405/488/532/635-t1 (Semrock, Rochester, NY, USA), respectively. The supracence from both 561 nm and 532 nm excitation was filtered by two respective ultra-sharp short-pass filters with >6 OD in wavelength longer than 561 nm, or 532 nm, respectively. Multi-edge dichroic mirrors are important because they allow corresponding supracence < 561 nm for rhodamine B and <532 nm for rhodamine 123, respectively, to pass through in high efficiency to the EMCCD detector (iXion+, ANDOR, South Windsor, CT, USA).

**Living cells labeled with spot or worm-like patterns**. In the imaging-quality, glass-bottom 35 mm MatTek’s culture dishes (MatTek Corp.) filled with Dulbecco’s Modified Eagle Medium (DMEM), breast cancer cells from a B16-F10 cell line were grown at 37 °C in a 5% CO_2_ incubator following manufacture protocol. Typically, at 70–80% confluence, we would treat the cells with nanoparticles containing rhodamine B to allow cells to endocytose these 40 nm particles. The solution nanoparticle concentrations are directly proportional to the level that living cells will endocytose. For example, we would dilute 3.3 μL of nanoparticle solution by 10X before mixing with 1 mL DMEM containing the above-cultured cells. After introducing the nanoparticles to the cells, the breast cancer cells were then incubated in the nanoparticle-containing medium for an additional 45 min to allow living cells to endocytose these labeled nanoparticles. To demonstrate super-spectral resolution, we would next use rhodamine 123 dye in solution to stain mitochondria. To avoid bias, the emission intensities of the two dyes should be as close as possible. For a typical example, 4.35 μL of 0.46 μM of rhodamine 123 in methanol solution would be diluted by 1 mL of the cell culture medium DMEM before adding to the above cell medium containing live cells with endocytosed nanoparticles. The breast cancer cells were then allowed to recover and attach to the glass bottom of the dish by further incubating in the combined medium for an additional 45 min. Then, the attached living cells were washed three times (2 mL each) with PBS buffer to remove dyes and nanoparticles in solution. The doubly stained living cells in the imaging buffer were transferred from the incubator to a homemade imaging device that was regulated at 37 ˚C via a constant-temperature fluid. The living cells that were stained with two closely emitting dyes were then imaged under various parameters specified in the discussions. Because the supracence signals were rather strong, the images reported here were acquired using a short exposure time of only 250 ms exposure time and normal CCD without EM gains.

## 4. Conclusions

This study has profound implications for future molecular science. The observation of molecular emission at wavelength shorter than excitation wavelength has eluded the community for quite some time and intuitively led to the direction of nonlinear excitation, i.e., multiple-step excitation by multiple energy sources: heat plus photon. Our experimental results demonstrated that heat is not a factor in supracence, and temperature has no effect on supracence intensity. The electron–atom quantum entanglement successfully predicts molecular quantum structural dynamics, transition-energy dynamics, and significantly, supracence. As a result, molecules do not have a solely energy-minimized structure, but rather a myriad of dynamic quantum structures that constantly exchange energy with molecular potential energy. Absorption and emission arrange into characteristic shapes defined by the quantum probability of the atoms; thus, they are not sticks; these predictions have been validated experimentally for decades but the electron–atom quantum interaction was not understood nor appreciated until now. 

This work not only solves a long-lasting problem of absorption and emission peak shape, but also underpins new phenomena such as supracence. Importantly, new molecular spectroscopy and microscopy of molecular supracence emerge under the quantum energy interactions. Of particular significance is that supracence has superior spectral resolution, thus imparting exciting technologies beyond current capabilities. The supracence imaging presented here indicates that eight different colors in a sample can be analyzed simultaneously, doubling current fluorescence capabilities. These breakthroughs will advance our knowledge in chemistry, expand capability in spectroscopy and molecular imaging, and spark imagination beyond current doctrines.

## 5. Patents

The authors were granted the US patent on supracence technologies.

## Supplementary Materials

The following supporting information can be downloaded at: https://www.mdpi.com/article/10.3390/ijms24087490/s1, Supplementary Information available: Movies of quantum structural dynamics and 2p_z_ orbital dancing in dual quantum model, dual-quantum model, and experimental section.
