# Supplementary material for: Full-Quantum Treatment of Molecular Systems Confirms Novel Supracence Photonic Properties
Source: Int J Mol Sci. 2023 Apr 19;24(8):7490. doi: 10.3390/ijms24087490 (PMC10138974; doi:10.3390/ijms24087490)
Supplement: Supplementary file 1 [file ijms-24-07490-s001.zip › mySupporting Information IJMS Revised.pdf]

Supporting Information for

Full-Quantum Treatment of Molecular Systems Confirms Novel Supracence Photonic Properties

Wei Wan, Alexander D. Q. Li*

Department of Chemistry, Washington State University, Pullman, Washington 99164, USA.

*Correspondence should be addressed: dequan@wsu.edu

Captions for Movies

Movie S1. The probability map created according to the heavier quantum atoms in perylene remain still in color gradients. Thus, each atom must orbit or "dance" according to the probability map. The atom position is revealed through the bond connections (white lines) and their positions shown in this movie are based on random picks correlated to their probability. Note this movie does not describe quantum motion, but rather to offer an understanding of quantum-quantum molecule instead of a traditional static or classical structure.

Movie S2. Because the lighter electronic quantum system is riding on the heavier atom quantum system, the electronic $2p_z$ orbitals, forming the conjugate π-structure that imparting visible emission of fluorescence and supracence, must also "dance" according to the tune generated by the quantum probability gradient of the atoms. The z-axis unit is in Angstrom (Å), but the x-y axis units are in 0.1 Å to enhance the dancing amplitudes of the electronic orbital.

Experimental Section

Supracence Spectrometer. Three models of supracence spectrometers were built: the first uses a tunable laser (SuperK, NKT Photonics), the second uses single-wavelength lasers, and the third uses 450 W Xe lamp through a double-monochromator. After the excitation light beam was conditioned to pure wavelength, it was focused (lamp) on the quartz sample cell. Supracence was measured using a PMT at a 90-degree angle like fluorescence. To ensure only the shorter wavelength or higher-energy photons were entering the detection monochromator, a high-quality short pass filter is placed in the front entrance of the detection monochromator when single-wavelength lasers are used. Laser excitations can result in very strong supracence. Thus, neutral density filters are usually placed in front of the detection monochromator to ensure the PMT is working within its linear response region.
